# Bowel function, urinary tract function, and health-related quality of life in males with anorectal malformations

**DOI:** 10.1007/s00383-024-05746-5

**Published:** 2024-06-27

**Authors:** Joshua Gertler, Anna Löf Granström, Jenny Oddsberg, Anna Gunnarsdóttir, Anna Svenningsson, Tomas Wester, Lisa Örtqvist

**Affiliations:** 1 Unit of Pediatric Surgery, Karolinska University Hospital, Astrid Lindgrens Barnsjukhus Karolinska Universitetssjukhuset, Eugeniavägen C11:33, Solna, 17176 Stockholm, Sweden; 2 Department of Women’s and Children’s Health, Karolinska Institutet, Stockholm, Sweden

**Keywords:** Anorectal malformation, Adulthood, Bowel function, Urinary tract function, QoL, Surgery

## Abstract

**Purpose:**

There is a knowledge gap regarding long-term outcomes for males undergoing surgery for an anorectal malformation (ARM). The purpose of this study was to investigate bowel function, bladder function, and health-related quality of life (HRQoL) in male patients with an anorectal malformation.

**Methods:**

This cross-sectional questionnaire-based study included males treated for ARM at our institution between 1994 and 2017. Bowel function was assessed with bowel function score (BFS) while urinary tract function was assessed with lower urinary tract symptoms (LUTS) questionnaires. Health-related quality of life (HRQoL) was investigated using age-relevant questionnaires (KIDSCREEN and PGWBI). Patient characteristics were retrospectively collected from the medical records and descriptive statistics were used for analysis. Functional outcomes were compared with gender and age-matched controls while HRQoL was compared to normative data. The study was approved by ethics review authorities.

**Results:**

A total of 58 (44.6%) of 130 males responded to the questionnaires. Regarding bowel function, 24 (42.1%) of 57 patients and 81 (95.3%) of 85 controls, respectively, reported a well-preserved bowel function represented by a BFS ≥ 17 (*p* < 0.001). Soiling issues and ‘feels urge’ items improved significantly with age. In a linear regression model, BFS increased significantly with age. For most parameters, the proportion of ARM patients with lower urinary tract symptoms was larger, though not significantly, compared to the controls. However, straining and stress incontinence were reported significantly more often by ARM patients. In patients and controls, voiding outcomes in terms of prevalence of having symptoms and the number of cumulative symptoms drop with increasing age. Children and adults reported similar or, in some domains, better HRQoL outcomes when compared to normative European data.

**Conclusion:**

Bowel function is impaired in male patients with ARM but significantly improves with age. Urinary tract function was affected, but overall comparable to the controls. HRQoL was unaffected. No significant association between the studied outcomes could be shown.

**Level of evidence:**

III.

**Supplementary Information:**

The online version contains supplementary material available at 10.1007/s00383-024-05746-5.

## Introduction

Congenital anorectal malformations (ARM) are one of many congenital anomalies among newborns. The birth prevalence of ARM in Sweden is approximately 1:3000 [1, 2]. There is a small predominance of male infants born with ARM with a sex ratio of 1.3:1 [1]. Associated malformations occurred in 50–67% patients with ARM in a registry-based study of 17 European regions [3]. It is assumed that associated malformations have a negative impact on overall outcomes of patients with ARM. Classification of ARM subtypes is essential to correctly treat patients and to facilitate comparative research in national and international settings. Previously, the Wingspread and Peña classifications were widely used. More recently, the clinically orientated Krickenbeck classification has been adopted [4–6]. Male anomalies include perineal cutaneous fistula, bulbar rectourethral and prostatic rectourethral, recto-bladder-neck vesical fistula, atresias without a fistula as well as anal stenosis. The vast majority of ARM patients require corrective surgery in the neonatal period or infancy. Peña and DeVries introduced the existing surgical techniques, a posterior sagittal ano-recto-plasty (PSARP), in the early 1980s [7]. Minimally invasive techniques using laparoscopic-assisted ano-recto-plasty (LAARP) now have a place in the management of selected male ARM patients [8]. The survival rates in infants with ARMs have steadily increased over the years alluding to the progress of surgical and neonatal care. As a result, a shift in treatment goals has occurred from survival to optimizing functional outcomes and preserving a good health-related quality of life (HRQoL). Composite and controlled data for outcomes of bowel function, urinary tract function, and quality of life are lacking. Among published work, quality of life and bowel function are shown to be impaired in patients with ARM [9–11]. Our group recently investigated these outcomes in female patients [12]. Here, we focus on male patients and aim to assess their outcomes into adulthood in a controlled study design.

## Methods

### Study design

This was cross-sectional questionnaire-based study. The study was registered in ClinicalTrials.gov (NCT04901819).

### Study setting

Individuals with ARM managed at the Unit of Pediatric Surgery at Karolinska University Hospital, Stockholm, Sweden. In 2024, Sweden has a population of roughly 10.6 million persons.

### Participants

All surgically managed males with ARM at our institution between 1994 and 2017 were eligible for the study. A database of identified eligible patients was created. The ARM subtypes which were included were perineal fistulas, rectourethral fistulas (both bulbar and prostatic), recto-bladder-neck fistulas as well as atresias without fistulas. Deceased patients and patients without surgical interventions were excluded from the study. In extension, patients with Currarino syndrome, Down’s syndrome, and patients with major intellectual disabilities were excluded from the study. After informed consent, participants and/or caregivers were asked to answer a composite questionnaire pertaining to the focus of study. Participants had the option to respond using paper mail or a digital platform (REDCap). A reminder was mailed to non-respondents after 4 and 8 weeks, respectively. Age categories (4–7, 8–12, 13–17, 18–26 years) determined which questionnaires were received. A control group of 2518 healthy age-matched individuals was randomly selected by Statistics Sweden from the Sweden Population Registry and invited to respond to Bowel function score (BFS) and Lower Urinary Tract Symptoms (LUTS) questionnaires. One hundred and ninety nine (8%) controls responded and 88 of them (44%) were males and thus used for comparison. Normative data were used to compare HRQoL outcomes [13].

### Data sources and variables

#### Patient characteristics

Patient characteristics and clinical details were recorded retrospectively from the medical records. These data included information about associated anomalies, ARM subtype according to Krickenbeck Classification, surgical procedures, and age at time of the study. The follow-up date was set to the 15th of June 2021.

#### Bowel function

Bowel function was assessed in all age categories using the previously validated BFS developed in the Finnish population [14, 15]. A BFS score of ≥ 17 of maximum 20 was used as an indicator of well-preserved bowel function as described previously by Kyrklund et al. [14]. Bowel function was evaluated in patients regardless of the use of laxatives, enemas, or antidiarrheal medication. No patients included in the cohort had enterostomies.

#### Urinary tract function

Urinary tract function was examined in all age categories with the validated 9-question lower urinary tract symptoms (LUTS) questionnaire [16]. Three out of seven items in the employed LUTS questionnaire were based on an adaptation from the previously validated Danish prostatic symptom score [17]. Patients with urinary diversion or who performed clean intermittent catheterization (CIC) were excluded prior to data analysis. Urinary tract function outcomes will be presented descriptively, the major endpoint being the prevalence of urinary incontinence defined as involuntary urinary leakage.

#### Health-related quality of life (HRQoL)

To assess children’s and adolescents’ (8–17 years old) subjective health and well-being, the KIDSCREEN-52 questionnaire was used as a validated instrument [18, 19]. The instrument covers 10 HRQoL dimensions encompassing physical well-being, psychological well-being, moods and emotions, self-perception, autonomy, parent relation and home life, financial resources, social support and peers, school environment, and social acceptance (bullying). The generic questionnaire is designed for both healthy and chronically ill children, also providing European normative data for comparison in this study [18]. In adults (18–26 years old), the validated instrument psychological general well-being index (PGWBI) was employed [13, 20, 21]. The instrument includes 6 dimensions comprising a total of 22 items with a maximal total score of 110, the higher the score the better HRQoL. The dimensions include anxiety, depressed mood, positive well-being, self-control, general health, and vitality. Outcomes of the survey are interpreted as follows; 0–60 “Severe Distress”, 61–71 “Moderate Distress”, 72–92 “No Distress”, and 93–110 “Positive Well-being” [21]. A score of roughly 80 is considered a mean score in large reference groups [22, 23]. Normative data for male healthy individuals were used when analyzing PGWBI outcomes.

### Statistical methods

Categorical variables were presented using frequencies and proportions, whereas continuous variables were presented as median with interquartile range (IQR) or mean with standard deviation (SD). LUTS and BFS values were compared between patients and age-matched female controls using the Fisher’s exact and Wilcoxon sum-rank tests, respectively. Trends between age groups and BFS item scores in the patient group were analyzed using asymptotic linear-by-linear association test. The overall BFS sum score was compared between age groups within cases and controls separately using non-parametric test (Jonckheere–Terpstra Test). For LUTS, a logistic regression model comparing patients and controls and adjusting for age as a continuous variable was designed to estimate the odds (OR) for having any LUTS (defined as presence of at least one symptom). KIDSCREEN values were translated to *T* values using the KIDSCREEN instruction manual and compared to European normative data for children & adolescents aged 8–17 years old using a *t*-test [10]. Likewise, observed PGWBI scores were compared to reference scores using a *t*-test [13]. The Spearman correlation test was used to analyze the association between BFS and the HRQoL variables (rho > 0.7 = strong correlation, > 0.4 moderate association, 0.2–0.4 = no correlation). *t*-tests were used to analyze relationships between the presence of LUTS and HRQoL variables. A significance level of *p* < 0.05 was used.

#### Ethical considerations

The study was approved by the Swedish ethical review authorities.

## Results

### Patient characteristics

**Fig. 1:**
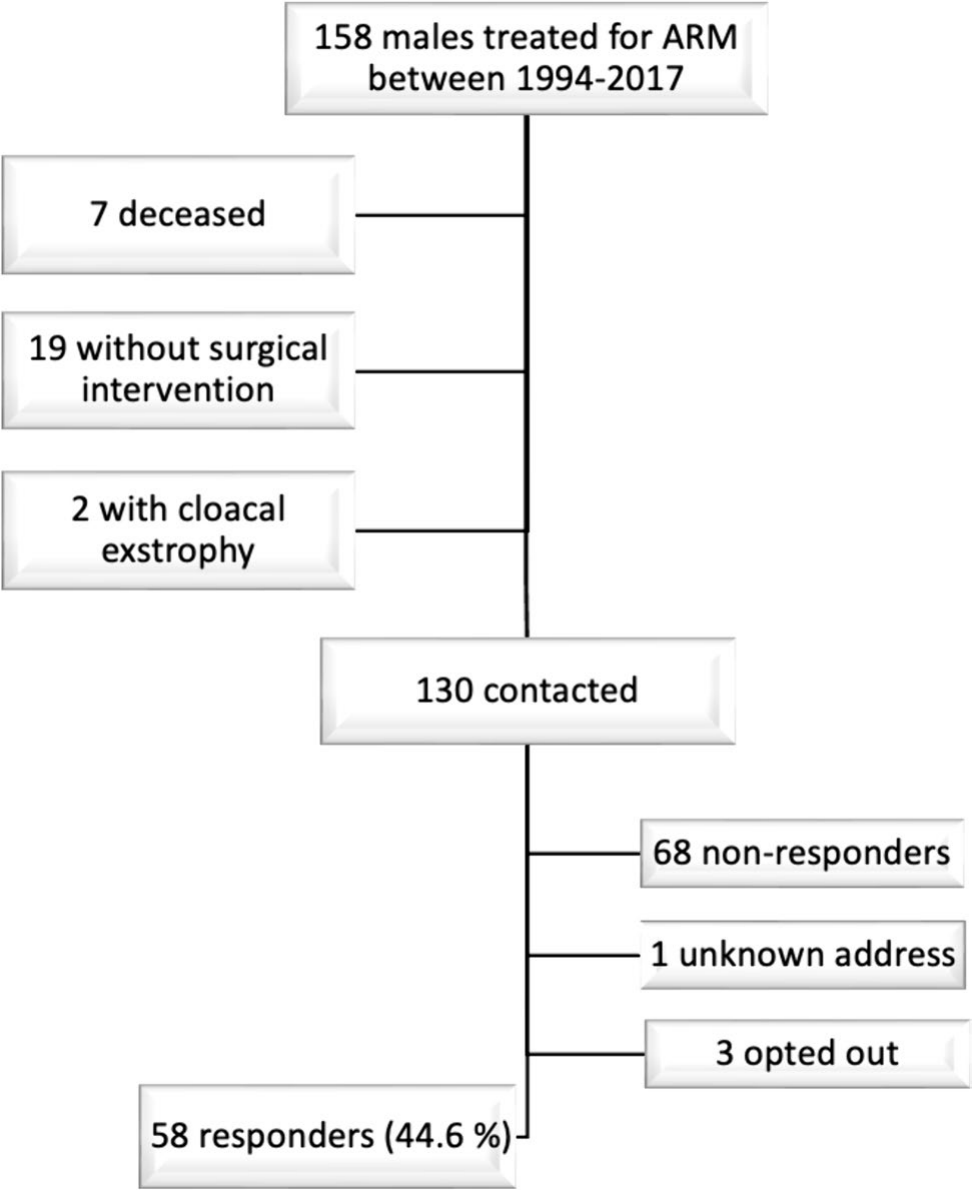
The participant’s inclusion process

The inclusion process is summarized in Fig. 1. The median age of the included patients and controls at the time of the study was 11 (IQR 7.8) years and 11.5 (IQR 8) years, respectively. One patient had Down syndrome and was thereby excluded from the study. Forty-six responders were 4–17 years of age and 11 were 18–26 years of age. The number of individuals included in each age group and their median ages are summarized in Supplementary Table 1. Thirty-four (59.6%) patients received a stoma neonatally. A PSARP was performed in 26/57 (45.6%) patients while a limited PSARP was employed in 25/57 (43.9%). One child and five adult patients had missing data concerning type of primary surgery. Forty percent of the patients had been treated for a perineal fistula and a just over a quarter (25.9%) had had a rectourethral fistula (Table 1). Due to lack of specificity in reporting in medical records, the rectobulbar and rectoprostatic urethral fistulas were pooled into one subgroup depicted as rectourethral fistulas. Associated malformations, for those included in the study, were common with 48.3% of patients having one or more associated malformation. VACTERL association was found in 15.5% of patients. Nine patients currently employed anterior continence enemas (ACE).

**Table 1:** Patient characteristics of the 58 responders

	*n* (%)	Missing data (*n*)
Median age at follow-up (years, median, IQR)	11 (7.75)	0
Type of ARM		7
Perineal fistula	23 (39.7)	
Rectourethral fistula	15 (25.9	
Atresia without fistula	7 (12.1)	
Recto-bladder-neck	3 (5.1)	
Other (e.g., stenosis)	3 (5.1)	
Associated malformations	28 (48.3)	5
Esophageal atresia	4 (6.9)	
Cardiac malformation	9 (15.5)	
Urinary tract anomalies	12 (20.7)	
Vertebral anomalies	12 (20.7)	
Tracheal anomalies	0	
Limb abnormalities	2 (3.4)	
VACTERL association	9 (15.5)	
Spinal cord abnormality	11 (19.0)	11
Permanent stoma at follow-up	0	
Antegrade continence enema (ACE)	8 (13.8)	
Permanent urinary diversion	2 (3.4)	
Current occupation among patients > 18 years, *n* = 12		
Student	6 (50.0)	
Full-time employed	3 (25.0)	
Part-time employed	4 (33.3)	
On sick leave	0	
Use of laxatives or enemas excluding ACE		
Children and adolescents	25 (54.3)	
Adults	5 (41.7)	

*IQR* Interquartile range, *ARM* Anorectal malformation, *ACE* Antegrade continence enema, *VACTERL* Vertebral-anal-cardiac-tracheo-esophageal-renal-limb

### Bowel function score

**Fig. 2:**
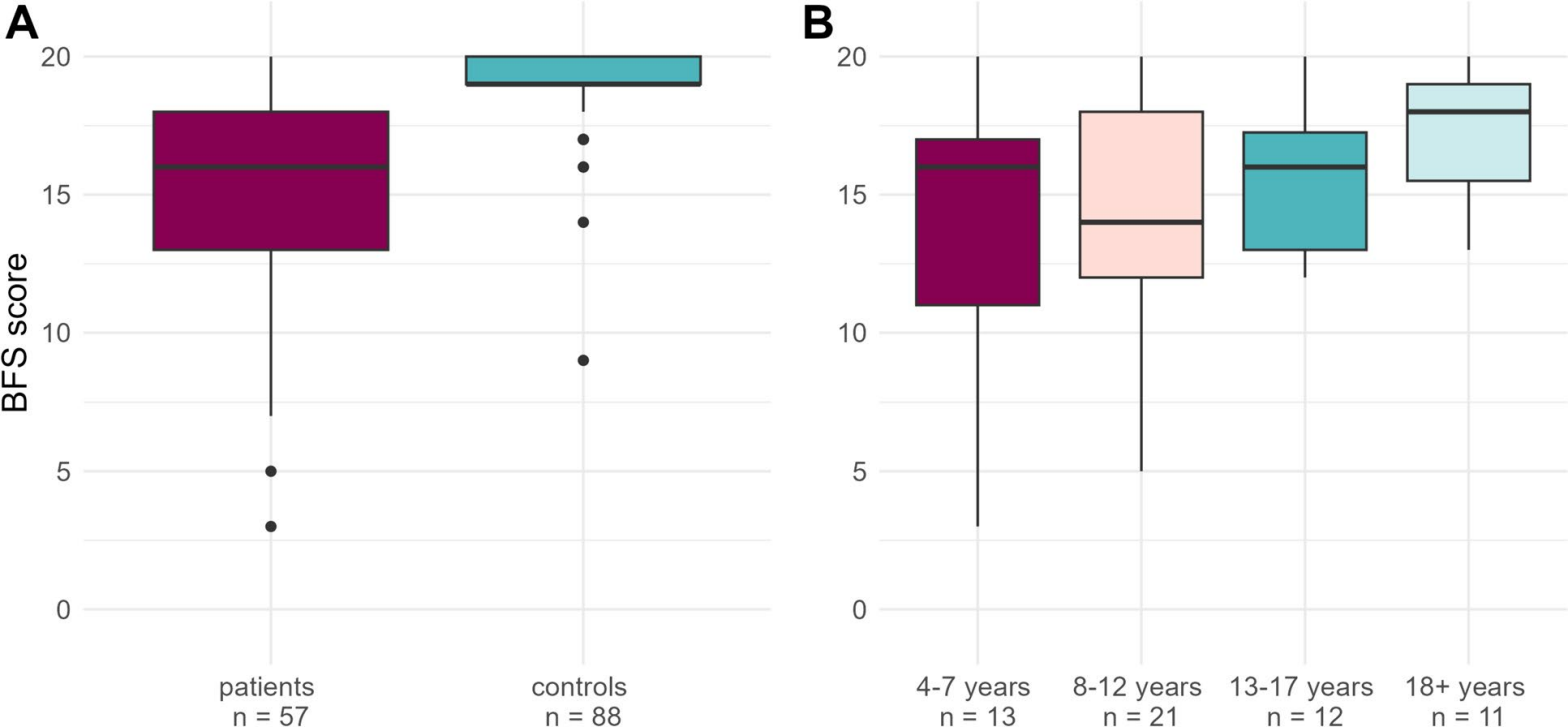
Median BFS scores for **A** patients vs. controls (*p* < 0.001) and **B** per patient age group (*p* = 0.034). *BFS* Bowel function score

**Fig. 3:**
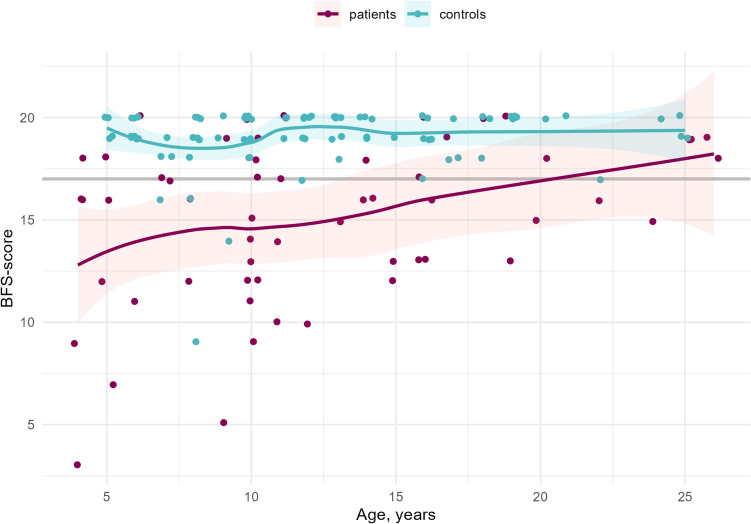
Scatter plot comparing BFS outcomes for patients (*p* = 0.008) and controls in relation to increasing age. *BFS* Bowel function score

No patients had a permanent enterostomy at the time of the study. Patients with ACE were included in the analysis. The median BFS for patients was 16 (IQR 5) compared to 19 (IQR 1) for controls (Fig. 2A) (*p* < 0.001). Due to missing values in the control responses, 85/88 controls could be used for data analysis. Differences in Median BFS scores between age groups within the cohort were statistically significant (Figs. 2B and 3). Both a non-parametric test (Jonckheere–Terpstra Test) and a linear regression model showed statistically significant increases in BFS with age, with *p*-values of 0.034 and 0.008, respectively. For every additional year of age, BFS is shown to increase 0.22 arbitrary units. A well-preserved bowel function defined as BFS ≥ 17 of 20 was found in 42.1% (24/57) of patients compared to 95.3% (81/85) in controls (*p* < 0.001) (Fischer’s exact test). In the patient cohort, 37.0% (17/46) of children and adolescents and 63.6% (7/11) of adults reported a well-preserved bowel function. The patients reported statistically significant lower scores across all items in the questionnaire when compared to the control group, except for their defecation frequency, displayed in Table 2.

**Table 2:** Bowel function scores per item and age groups, comparing cohort and controls

	Item score	Cohort, *n* (%)	Controls, *n* (%)	*p*-value*
*n*		57	88	
Age, mean (SD)		12.33 (6.13)	12.15 (5.28)	0.846
Age group				
4–7 years		13 (22.8)	19 (21.6)	0.957
8–12 years		21 (36.8)	32 (36.4)	
13–17 years		12 (21.1)	22 (25.0)	
18–26 years		11 (19.3)	15 (17.0)	
Feels/reports the urge to defecate	0	2 ( 3.5)	1 (1.1)	< 0.001
	1	10 (17.5)	1 (1.1)	
	2	11 (19.3)	5 ( 5.7)	
	3	34 (59.6)	81 (92.0)	
Ability to hold back defecation	0	6 (10.5)	1 (1.1)	< 0.001
	1	8 (14.0)	1 (1.1)	
	2	15 (26.3)	3 (3.4)	
	3	28 (49.1)	83 (94.3)	
Frequency of defecation	1	13 (22.8)	12 (13.8)	0.182
	2	44 (77.2)	75 (86.2)	
Soiling	0	4 ( 7.0)	0 ( 0.0)	< 0.001
	1	11 (19.3)	2 ( 2.3)	
	2	22 (38.6)	21 (24.1)	
	3	20 (35.1)	64 (73.6)	
Accidents	0	1 ( 1.8)	0 ( 0.0)	< 0.001
	1	4 ( 7.0)	0 ( 0.0)	
	2	11 (19.3)	4 ( 4.5)	
	3	41 (71.9)	84 (95.5)	
Constipation	0	10 (17.5)	1 ( 1.1)	< 0.001
	1	16 (28.1)	2 ( 2.3)	
	2	10 (17.5)	11 (12.6)	
	3	21 (36.8)	73 (83.9)	
Social problems	0	2 ( 3.5)	0 ( 0.0)	< 0.001
	1	7 (12.3)	1 ( 1.1)	
	2	8 (14.0)	1 ( 1.1)	
	3	40 (70.2)	86 (97.7)	
BFS score (median [IQR])		16.00 [13.00, 18.00]	19.00 [19.00, 20.00]	< 0.001
BFS ≥ 17/20, *n* (%)	0–16	33 (57.9)	4 ( 4.7)	< 0.001
	17–20	24 (42.1)	81 (95.3)	

*IQR* Interquartile range, *BFS* Bowel function score

^*^Fisher’s exact test

A linear-by-linear asymptotic association test was used and suggested statistically significant trends in 2 of the 7 BFS-items. A positive trend was seen in the ‘feels urge’ item where it improved with increasing age group (*p* = 0.026). Further, a positive trend was seen in ‘soiling’ where this issue mitigated with increasing age group (*p* = 0.001). No other significant trends were identified relating to individual BFS items and age.

### Urinary tract function

One child and one adult, both having sacral dysgenesis, used CIC and were excluded from this analysis. The sense of urgency and the need to strain were the most common symptoms in the cohort with 17.2% and 16.3%, respectively, having these symptoms to some degree. For the controls, bedwetting was the most common symptom reported with a percentage of 11.3. Fisher’s exact test was performed to compare age group distribution and overall LUTS prevalence between patient and controls. No statistical differences (*p* = 0.975 and *p* = 0.299, respectively) were observed. However, the estimated odds ratio for having LUTS (LUTS > 0) as a patient was 1.8 times higher than for controls (95% CI 0.85, 3.83). Stress incontinence and straining during urination were significantly higher in the cohort (*p* = 0.031 and 0.007, respectively). Bedwetting in the cohort was reported marginally more frequently, *p* = 0.050. Including spontaneous leakage, as a measure of voiding incontinence, no other factors were significantly different between patients and controls. No adult patients in the cohort reported stress incontinence, urge incontinence, bedwetting, social problems due to incontinence, or spontaneous leakage. In patients and controls, both the prevalence of having symptoms and the number of cumulative symptoms drop with increasing age. A logistic regression model using age as a continuous variable showed that the odds ratio (OR) decreases for LUTS 0.87 times (95% CI 0.80, 0.93) for every additional year of age (*p* < 0.001).

### HRQoL

#### KIDSCREEN for children and adolescents

**Fig. 4:**
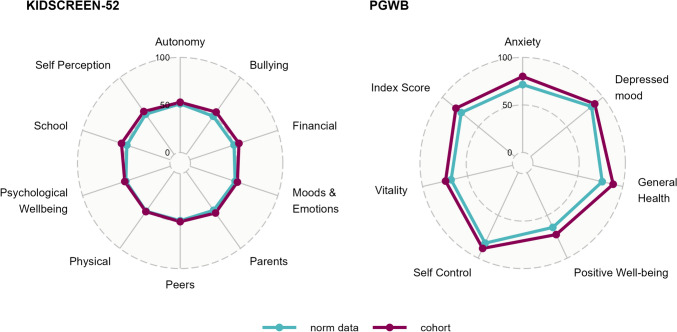
Radial diagram of KIDSCREEN-52 and PGWBI cohort mean values per domain compared to normative data. *PGWBI* Psychological general well-being index

Visually, the normalized mean domain *t*-values in the cohort did not differ significantly from the age- and gender-matched European normative data (Fig. 4). Paradoxically, cohort patients had a significantly higher score in the financial resources, parent relations, school environment, and bullying social acceptance domains (*p* = 0.000, 0.026, 0.002, 0.001, respectively).

#### PGWBI for adults

The mean normalized PGWBI for the cohort was 81.3 compared to the reference data having a mean score of 73.9 (*p* = 0.002). Table 3 summarizes the cohort and normative data per domain. The cohort scored significantly higher in several domains including ‘Anxiety’ and ‘General Health’. The cohort did not score inferiorly to the norm data in any domain, graphically visualized in Fig. 4. One patient scored within the “severe distress” bracket (0–60 points). This patient also utilized CIC and had the poorest BFS (13/20) of the adult group. 8 patients (72.7%) had “no distress” (72–92) and 2 patients (18.2%) reported “being positive” (72–110). None of the adult patients were on sick leave at follow-up time and 90.9% of them did not subjectively feel that their choice of occupation was affected by their ARM (Table 1).

**Table 3:** PGWBI domain scores compared to norm data

PGWBI domain	Cohort, mean (SD)	Normative data, mean (SD)	*p*-value
Anxiety	80.0 (9.6)	71.4 (19.3)	0.020
Depressed mood	88.7 (7.7)	84.2 (16.7)	0.101
General health	89.3 (8.4)	77.2 (19.2)	0.001
Positive well-being	72.5(12.7)	64.1 (18.9)	0.067
Self-control	88.7(9.5)	82.5 (17.6)	0.069
Vitality	74.5(10.1)	68.3 (18.9)	0.085
Index score	81.3 (5.2)	73.9 (15.5)	0.002

*PGWBI* Psychological general well-being index

### Composite outcomes

#### KIDSCREEN and BFS

A Spearman correlation test (Rho = *p*) was performed to evaluate correlation between KIDSCREEN item t scores and BFS. Overall, no correlations could be found except for the patient’s autonomy (*p* = 0.525, > 0.4 = moderate correlation) and financial aspects (*p* = 0.442). There is therein a moderate association between a higher BFS and better subjective autonomy and their perceived financial situation.

#### KIDSCREEN and LUTS

Assuming that KIDSCREEN *T *values have a normal distribution, a *t*-test was used to see if a relation was present between KIDSCREEN items and the presence of LUTS (excluding history of UTI). No associations were found.

#### PGWBI, LUTS, and BFS

The relationships of PGWBI contra LUTS and BFS were analyzed. However, no associations could be shown.

#### ARM subtype and BFS

The subtype groups for perineal (*n* = 21) and rectourethral (*n* = 14) fistulas in children and adolescents were large enough to analyze where 62% and 14% had a BFS ≥ 17/20, respectively (exact test *p* = 0.007).

## Discussion

### Key findings

The bowel function of males treated for ARM was grossly impaired across all age groups when compared to healthy controls. For the cohort, 42.1% reported an acceptable bowel function compared to 95.3% in the controls. Bowel function in the cohort improved with 0.22 units per year of age and 63.6% of adult patients reported a BFS of ≥ 17/20. Soiling issues and the ability to feel the urge to defecate improved significantly in the cohort with increasing age group. Contrarily to findings by Rintala et al. and our previous study on females, constipation issues did not change with increasing age [12, 24]. Voiding outcomes in general were comparable to the controls. Nonetheless, LUTS were more common in the ARM group compared to the controls. The OR decreases for LUTS 0.87 times for every additional year of age. The sense of urgency and the need to strain to urinate were the most common LUTS in the cohort. Involuntary leakage did not occur in any of the adult patients in the study. In accordance with a recent systematic review showing a high LUTS prevalence (36%) 10 years after surgical repair, 41.8% of patients in our cohort had at least one LUTS [25]. Our data is in the middle range of what has been reported in the literature (18–72%) [25, 26]. The recent review article describes a UTI rate of 36.4% compared to 13% in our cohort [26]. In accordance with previous results, HRQoL was preserved with children and adolescents in this cohort [12, 27]. Surprisingly, the cohort scored significantly higher in the domains of financial resources, parent relations, school environment and bullying social acceptance. Further, an association was found between BFS and the children’s feelings of autonomy and their financial perceptions. Regarding autonomy, this relation was recently described even for female ARM patients in our previous study [12]. Hypothetically, an acceptable bowel function could influence autonomy, not having to ask for help or assistance if, for example, fecal accidents happen. In the adult group, the HRQoL was preserved and paradoxically scored higher on the overall score when compared to normative data. Nearly three-quarters of patients reported “no distress” which is the same category where the mean result (80) is found in large reference groups [23]. The PGWBI scores could not be associated statistically to either LUTS or BFS.

### Interpretation

Through literature review, few studies have focused on gender-specific outcomes after surgical repair of ARM while comparing them to matched controls. In extension to our previous research, “partially pertaining to anatomical differences, the phenotypes of the malformations differ between males and females and thus should be studied separately” [12]. Studying the genders separately has suggested major differences in the two groups. In males, BFS and LUTS improved with increasing age and even adult patients had a preserved HRQoL. This was not the case for our female cohort [12]. Choosing a suitable questionnaire was crucial for this study. The multivariate BFS by Rintala et al. was employed to facilitate comparison of outcomes between present and future ARM studies [15]. Through literature review, BFS has been trending in recent years. Further, geographically neighboring Nordic countries, having similar sociocultural–economic structures and health care systems are deemed to be comparable on the population general health level [14]. This study confirms previously published results reporting bowel function impairment in ARM patients [28]. In this study, 42.1% of patients had acceptable bowel function, a figure substantially higher than what we previously reported for females (32.6%) from the same center and time period [12]. This points to the heterogeneity of the malformation and gender-specific differences. A mean BFS of 15.1 (SD 4.0) in our study is comparable to 13.9 found by Kaselas et al. although Kaselas pooled females and males together. ARM subtype is of interest in relation to BFS. However, our cohort was too small to yield substantial power for most subgroup analyses. However, we found that perineal fistulas in children had better bowel function outcomes compared to children with recto-urethral fistulas. In 2005, Levitt and Peña concluded that constipation was the most common complication in patients who had undergone PSARP [29]. Second to soiling issues reported in 64.9% of our cohort, 63.2% of patients had some degree of constipation. Interpreting HRQoL of patients with congenital malformations is a challenge. For example, these patients were born with their condition and thereby their reference of HRQoL could arguably differ from that of the reference population. In children and adolescents in this study, their reported HRQoL was not inferior to the normalized reference data. In fact, our cohort scored higher on several items. These findings are on par with results reported by Wigander et al. from a Swedish cohort of children with low ARM where patients and controls had comparable HRQoL (HAQL tool) [30]. A recently published paper by Beattie et al. found conflicting results to ours where they used the PedsQL tool showing significantly impaired quality of life in children [31]. Further, Beattie et al. reported no differences in QoL between genders in children with ARM. HRQoL studies performed by Hartmann et al. show a large variation in quality of life in patients treated for ARM [32]. Patients doing very poorly are an urge for concern and must be identified early on by clinicians to implement extra care [33]. Örtqvist et al. recently reported in a multi-center Nordic study that even patients operated on for a cloacal malformation had similar HRQoL compared to healthy a Swedish population [34]. Critical comparison of studies is made difficult due to study design differences as well as the use of different investigating tools. Few studies have focused on the span of ages into adulthood for males regarding HRQoL and there lies no consensus for which HRQoL tool should be used. For our purposes, the KIDSCREEN tool was suitable as it has been broadly used in the Nordic setting and has normative data on a large scale.

In adult males from this cohort, their HRQoL was not either found be inferior to normative European data. Published work on this topic with similar cohorts is scanty. A thirty-year-old paper by Hassink et al. looking at 58 adults having been operated for a high ARM observed that “most aspects of QoL and mental health did not differ from those of the general population” [35]. It could be speculated that coming into puberty and adulthood leading to sexual debut could lead to impaired HRQoL. However, this does not seem to be the case in our cohort. Composite outcomes in this setting have been scantily studied to this point. We aimed to analyze potential associations between HRQoL, bowel function and urinary tract function. Parallelly to the previously published female cohort, BFS is even shown to be associated to the feeling of autonomy in male children [12]. Further, male children and adolescents’ BFS correlates moderately to the financial resources item of HRQoL. These aspects are assumed to be influenced by several variables which is why the associations found were moderate. Children with perineal fistulas statistically had better bowel function outcomes than children with rectourethral fistulas. This is on par with previous research where higher, more complex malformations have been shown to have impaired bowel continence [31, 36, 37].

In general, the relatively small groups of patients and large number of variables make these analyses challenging. Furthermore, certain analyses which had been envisioned were unable to be carried out. For example, we collected data on spinal and other associated anomalies. However, the amount of missing data proved to be too large to yield generalizable results (data not published). Overall, the same applies for the influence that ARM subtype might have on BFS, LUTS, and HRQoL.

### Limitations

To reduce confounding factors, the heterogenicity of the cohort was limited. Selection bias of the responders was minimal as potential participants met the inclusion criteria before asked to answer the questionnaires. Yet, it is possible that patients in need of medical attention have a greater inclination to participate which would create a selection bias. A first limitation was the sample size. Our intended secondary aim to examine long-term outcomes per ARM subtype proved difficult to answer due to the low response rate (44.6%) subsequently making subgroups too small to generate statistical power concerning certain issues. Additionally, the response rate in the control group was likewise low. A second limitation involves the presence of ongoing treatments with laxatives and, or antidiarrheal medications, which the healthy controls were not exposed to. A third and final limitation is the cross-sectional study design. Not having followed a specific patient over time, it is difficult to evaluate changes in outcomes over time. For instance, we cannot conclude that a specific ARM patients’ BFS improves with age, although there is a general difference between age groups. We can, however, indicate that even LUTS prevalence decreases with increasing age and that HRQoL is preserved in all ages. Another factor potentially influencing outcomes is the heterogenicity of ARM subtype complexity per age group. As the number of individuals with a specific subtype varied per age group, this could represent a confounding factor when interpreting results. A causality relationship between HRQoL and the congenital malformation could not be shown in this study. HRQoL is multifactorial and the response-shift umbrella concept should always be considered when interpreting HRQoL data [38].

## Conclusion

Roughly 40% of the male ARM patients reported well-preserved bowel function, a function that improved with age. LUTS prevalence diminished with age and no adults had involuntary urinary leakage. However, it must be noted that patients using CIC were excluded. Neither children nor adults were found to have an inferior HRQoL when compared to norm European data. Composite variable analysis proved difficult. These findings confirm the importance of transitioning male adolescents to adult care that can provide expertise in the sequelae of anorectal malformations.

## Supplementary Information

Below is the link to the electronic supplementary material.

Supplementary file1 (DOCX 14 KB)

## Data Availability

No datasets were generated or analyzed during the current study.

## Abbreviations

ACE: Antegrade continence enema
ARM: Anorectal malformation
BFS: Bowel function score
CI: Confidence interval
CIC: Clean intermittent catheterization
HRQoL: Health-related quality of life
IQR: Interquartile range
PGWBI: Psychological general well-being index
PSARP: Posterior sagittal ano-recto-plasty
LAARP: Laparoscopically assisted ano-recto-plasty
LUTS: Lower urinary tract symptoms
SD: Standard deviation
UTI: Urinary tract infection
VACTERL: Vertebral-anal-cardiac-tracheo-esophageal-renal-limb
