# Supplementary material for: Bowel function, urinary tract function, and health-related quality of life in males with anorectal malformations
Source: Pediatr Surg Int. 2024 Jun 27;40(1):164. doi: 10.1007/s00383-024-05746-5 (PMC11211194; doi:10.1007/s00383-024-05746-5)
Supplement: Supplementary file 1 — Supplementary file1 (DOCX 14 KB) [file 383_2024_5746_MOESM1_ESM.docx]

**Supplementary Material:**

| Age Group, y | Cohort, n (%) | Controls, n (%) |
| --- | --- | --- |
| 4-7 | 13 (22.8) | 19 (21.6) |
| 8-12 | 21 (36.8) | 32 (36.4) |
| 13-17 | 12 (21.1) | 22 (25.0) |
| 18+ | 11 (19.3) | 15 (17.0) |

**Supplementary table 1:** Sample sizes of cohort and controls age groups.
